# Magnolol Ameliorates Behavioral Impairments and Neuropathology in a Transgenic Mouse Model of Alzheimer's Disease

**DOI:** 10.1155/2020/5920476

**Published:** 2020-07-03

**Authors:** Yan-Fang Xian, Chang Qu, Yue Liu, Siu-Po Ip, Qiu-Ju Yuan, Wen Yang, Zhi-Xiu Lin

**Affiliations:** ^1^School of Chinese Medicine, Faculty of Medicine, The Chinese University of Hong Kong, Shatin, N.T., Hong Kong SAR, China; ^2^Brain Research Centre, School of Chinese Medicine, Faculty of Medicine, The Chinese University of Hong Kong, Hong Kong SAR, China; ^3^Cardiovascular Disease Centre, Xiyuan Hospital of China Academy of Chinese Medical Sciences, Beijing, China; ^4^Hong Kong Institute of Integrative Medicine, The Chinese University of Hong Kong, Hong Kong SAR, China

## Abstract

Alzheimer's disease (AD) is a common neurodegenerative disease characterized by progressive memory loss. Magnolol (MN), the main active ingredient of *Magnolia officinalis*, possesses anti-AD effects in several experimental models of AD. In this study, we aimed to explore whether MN could ameliorate the cognitive deficits in TgCRND8 transgenic mice and to elucidate its molecular mechanisms. Male TgCRND8 mice were orally administered with MN (20 and 40 mg/kg) daily for 4 consecutive months, followed by assessing the spatial learning and memory functions using the open-field, radial arm maze, and novel object recognition tests. The results demonstrated that MN (20 and 40 mg/kg) could markedly ameliorate the cognitive deficits in TgCRND8 mice. In addition, MN significantly increased the expression of postsynaptic density protein 93 (PSD93), PSD-95, synapsin-1, synaptotagmin-1, synaptophysin (SYN), and interleukin-10 (IL-10), while markedly reduced the protein levels of tumor necrosis factor alpha (TNF-*α*), IL-6, IL-1*β*, A*β*_40_, and A*β*_42_, and modulated the amyloid precursor protein (APP) processing and phosphorylation. Immunofluorescence showed that MN significantly suppressed the activation of microglia (Iba-1) and astrocytes (GFAP) in the hippocampus and cerebral cortex of TgCRND8 mice. Mechanistic studies revealed that MN could significantly increase the ratios of p-GSK-3*β* (Ser9)/GSK-3*β*, p-Akt (Ser473)/Akt, and p-NF-*κ*B p65/NF-*κ*B p65. These findings indicate that MN exerted cognitive deficits improving effects via suppressing neuroinflammation, amyloid pathology, and synaptic dysfunction through regulating the PI3K/Akt/GSK-3*β* and NF-*κ*B pathways, suggesting that MN is a promising naturally occurring polyphenol worthy of further developing into a therapeutic agent for AD treatment.

## 1. Introduction

Alzheimer's disease (AD), the most common type of dementia in the elderly population, is characterized by progressive memory loss and cognitive decline [1, 2]. AD affected about 26.6 million people worldwide in 2006, and the number is expected to rise to 131.5 million by 2050 [3]. The healthcare and economic burden due to AD to the society is enormous [4]. Pathologically, the extracellular beta-amyloid plaque (A*β*) deposits composed of A*β* peptides and the intracellular neurofibrillary tangles as a result of tau protein accumulation in the brain are the two major hallmarks of AD. Accumulating evidence suggests that neuroinflammation and the loss of neuronal synapses are observed in the early stage of AD and are associated with cognitive decline [5]. The underlying mechanisms of the onset and progression of AD are still unclear. Mutation of the amyloid precursor protein (APP) induces the abnormal production of A*β* peptides by *β*- and *γ*-secretase and is believed to play a critical role in the early onset of familial AD [6]. In the APP processing, beta-site APP-cleaving enzyme 1 (BACE-1), the major *β*-secretase enzyme, is directly involved in the cleavage of APP. Neurons of BACE-1−/− mice do not generate A*β*, suggesting that BACE-1 is the neuronal *β*-secretase [7]. Phosphorylated APP at the Thr668 site (p-APP (T668)) can facilitate the APP processing at the *β*-cleavage site. Presenilin 1 (PS1) and anterior pharynx-defective 1 (APH-1) are the two major components of *γ*-secretase [8]. Neprilysin (NEP) and insulin-degrading enzyme (IDE) are the two major A*β*-degrading enzymes that promote A*β* degradation [9].

The TgCRND8 transgenic mouse, a well-known aggressive APP mouse model of AD, expresses the human APP gene with double KM670/671NL+V717F Swedish and Indiana familial AD mutations. TgCRND8 mice overexpress mutant human APP at a level approximately 5-fold higher than endogenous murine APP. TgCRND8 mice express extracellular A*β*40 and A*β*42 at one month of age, and the soluble A*β*42/A*β*40 ratios are found to be elevated at two months of age [10]. At 3 months of age, TgCRND8 mice develop the phenotypes closely resembling human AD such as A*β* deposits, astrocytic activation, microglial activation, neuritic dystrophy, inflammation, and learning and memory deficits [11, 12]. Therefore, the TgCRND8 mouse model is a valuable tool for investigating new therapeutic agents for AD and elucidating the underlying anti-AD molecular mechanisms.

**Figure 1:**
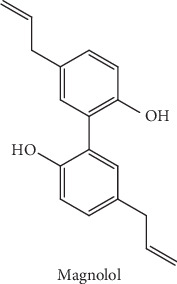
Chemical structure of magnolol (MN).

Currently available drugs for AD can only ameliorate symptoms of AD but fall short of reversing or even slowing down the disease progression. Therefore, therapeutic strategies for thwarting AD progression clearly remain an unmet medical need. Magnolol (MN) (the chemical structure is shown in Figure 1) is the essential natural neolignan and the main active ingredient responsible for the therapeutic properties of the bark of *Magnolia officinalis*, a herb widely used in Chinese medicine to treat inflammatory diseases with low toxicity [13]. The content of MN in the bark of *M. officinalis* is about 1.0-1.25% [13]. MN has been shown to exert various pharmacological activities such as anti-inflammation [14], antioxidation [15], and neuroprotection [16, 17]. MN has recently been reported to possess anti-AD effects in experimental models of AD [18–20]. MN significantly alleviates the A*β*-induced neurotoxicity via suppressing the intracellular calcium elevation, the reactive oxygen species production, the caspase-3 activity, and inflammation, as well as promoting the phagocytosis and degradation of A*β* [18–20]. In addition, MN has been shown to prevent the cognitive deficits induced by scopolamine in mice via inhibition of the acetylcholinesterase activity and oxidative stress [19]. Moreover, MN has been demonstrated to ameliorate learning and memory impairments by preserving cholinergic function in the forebrain of the SAMP8 mice [17]. Importantly, MN could cross the blood-brain barrier (BBB) and remain relatively stable in the brain after oral administration [21]. Moreover, no troublesome side effects have been reported so far in humans after ingestion of MN [13]. All these observations indicate that MN may be the active principle responsible for the anti-AD activity of *M. officinalis*. However, the molecular mechanisms underlying the anti-AD actions of MN hitherto remain unexplored. In the present study, we aimed to investigate whether MN could ameliorate the learning and memory impairments in TgCRND8 transgenic mice and illustrate its mechanisms of action.

## 2. Materials and Methods

### 2.1. Chemicals and Reagents

Magnolol (MN, purity ≥ 98%) was purchased from Hong Kong University of Science and Technology Research and Development Corporation Limited. Its identity was confirmed by comparing its ^1^H NMR and ^13^C NMR spectra with that published in the literature [22]. A*β*_42_ peptide was purchased from GL Biochem Ltd. (Shanghai, China). Donepezil hydrochloride was purchased from Sigma-Aldrich (St. Louis, MO, USA). All other chemicals and reagents used in this study were of analytical grade.

### 2.2. Animals

TgCRND8 mice harbor the genetic background of (C57BL/6J) × (C3H/HeJ × C57BL/6J). Male TgCRND8 mice and female wild-type C57BL/6J were used to breed a colony of experimental animals. Nontransgenic littermates that did not express human APP transgene were identified as wild-type mice and used as a control group. The mice were bred in the Run Run Shaw Science Building, The Chinese University of Hong Kong, and routinely maintained on a 12 h light/dark cycle under controlled humidity (50 ± 10%) and temperature (22 ± 2°C) with access to food and water *ad libitum*.

### 2.3. Genotyping of TgCRND8 Mice

In order to genotype the APP transgene, DNA was extracted from the ear tissues of all mice. The APP transgene was determined by a transgene-specific PCR reaction using the following primers: forwards: TGTCCAAGATGCAGCAGAACGGCTAC, reverse: GGCCGCGGAGAAATGAAGAAACGCCA. Briefly, a visible amount of ear tissue was digested in the non-SDS tissue digesting buffer (500 mM KCl, 100 mM Tris-HCl, 0.45% NP-40 (Igepal™ CA-630), 0.1 mg/ml gelatin, and 0.45% Tween 20) with proteinase K (Cat. V900887, Sigma) at 55°C overnight. After heated at 98°C for 10 min to inactivate proteinase K, the supernatant was collected after centrifugation and then undergone a PCR reaction with a TaKaRa Taq™ package (Cat. R001A, TaKaRa) and the primers. The reactions were run at 95°C for 5 min, followed by 45 cycles at 95°C for 30 s, 54°C for 40 s, 72°C for 80 s, and 72°C for 10 min. The PCR samples were separated using 1% agarose gel, then observed under UV light for 30 s. The mice with APP transgene were identified as transgenic mice, while those without APP transgene as wild-type mice.

### 2.4. Grouping of TgCRND8 Mice and Drug Treatment

**Figure 2:**
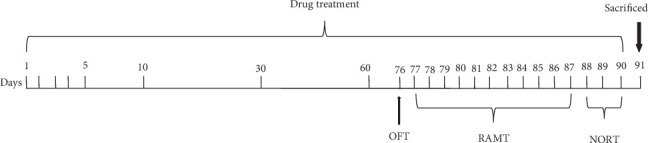
Experimental design and treatment schedule.

Three-month-old male mice were randomly assigned to five groups of 10 animal each: (a) wild type (WT), (b) TgCRND8 (Tg)+vehicle, (c) Tg+MN (20 mg/kg), (d) Tg+MN (40 mg/kg), and (e) Tg+donepezil (5 mg/kg). The dosages of MN used in this study were chosen based on our pilot study (data not shown). Donepezil was chosen as a positive control based on previous publications [23, 24]. MN was suspended in 0.5% sodium carboxymethyl cellulose (CMC-Na) while donepezil was dissolved in normal saline. Mice were administered orally with MN and donepezil by gavage once daily for 4 months, whereas the mice in the WT group and the Tg+vehicle group received an equal volume of 0.5% CMC-Na. After drug treatment, the spatial learning and memory functions were assessed using the open-field test, the novel objective recognition (NOR) test, and the radial arm maze (RAM) test.  Figure 2 shows the experimental design and schedule.

### 2.5. Open-Field Test (OFT)

The locomotor activity of the mice was determined by the OFT. Briefly, the mice were placed in an open field (40 × 60 × 50 cm) with a brown floor divided into 12 equal squares and a frontal glass wall [25]. The mice were subjected to two identical sessions on two consecutive days, with the first session for training and the second one for testing. Each session lasted for 6 min. The number of line crossing with four paws and the number of rearing (number of times the animals stood on their hind legs) were recorded to investigate the exploratory behavior and locomotor activity of mice, respectively, by two observers who were blinded to the grouping information. To avoid perturbation to the animals due to urine and feces, the apparatus was cleaned with 10% ethanol solution and a piece of dry cloth between two tests.

### 2.6. Novel Object Recognition Test (NORT)

The NORT were conducted in an open-field arena (30 × 30 × 30 cm) constructed with polyvinyl chloride, plywood, and acrylic as previously described [26]. The tasks included a training session and a recognition session for two consecutive days. On day 1, the mice were allowed to explore two identical objects (5 × 5 × 5 cm, blue plastic cubes) for 5 min in the training session. On day 2, one of the objects was replaced with a new shape and color (5 × 5 × 7 cm, a white plastic square pyramid), and the mice were acclimatized to the area for 5 min in the recognition session. The fields were decontaminated with a 10% ethanol solution between the tests. The animals were allowed to explore the test area by touching or sniffing the objects with their forepaws and/or noses at a distance of less than 2 cm. The total exploration time was the amount of time devoted to locating the two objects. The time of each mouse spent exploring the objects was recorded by two investigators who were blinded to the experimental design. The cognitive function was determined using a recognition index, which was the exploration time involved with either of the two objects (training session) or the novel object (recognition session) divided by the total exploration time in exploring both objects.

### 2.7. Radial Arm Maze Test (RAMT)

The spatial learning and memory functions of mice were determined using the RAMT. The apparatus for the RAMT was obtained from Xinruan Information Technology Co. Ltd. (Xinruan, Shanghai, China) with a video tracking software of SuperMaze V2.0. The apparatus comprises eight radial arms (10 cm high, 5 cm wide, and 35 cm long) numbered from 1 to 8 and a central platform (22 cm in diameter). The RAMT was conducted as described in our previous studies [24, 27]. During the behavioral test period, to stimulate hunger, the mice were maintained on a restricted diet with only water being available *ad libitum.* The body weight of mice was kept at 85-90% of free-feeding level. The RAMT lasted for 8 consecutive days: 2 days for habituation trials, 5 days for training trials, and 1 day for task test. At the habituation trial, 3 or 4 mice were simultaneously put in the central platform of RAM, and all arms were baited with several food pellets about 10 mg each. After two days of habituation trial, the mice were trained with 1 trial daily for 5 consecutive days. At the training trial, only 4 constant arms were baited with one food pellet about 10 mg, which was placed in the nontransparent food cup to prevent visual detection, and only one mouse was placed in the central platform. The mice were trained to run to the end of the baited arms and consume all the food pellets within 10 min. The mice were subjected to working and reference memory task tests on the eighth day. In the task test, the same four arms were baited with one food pellet about 10 mg, and an arm entry was counted when all four limbs of the mice were within an arm. After all of the food pellets had been consumed or 10 min had passed, the task test was completed. In the task test, two observers who were blinded to the grouping information recorded the following data: (1) the number of working memory errors (WMEs), which meant the reentries into an already visited baited arm during the period of task test; (2) the number of reference memory errors (RMEs), which meant the entries into the nonbaited arms during the period of task test; and (3) the number of total entries to complete the task test.

### 2.8. Brain Tissue Collection

Twenty-four hours after the NORT, the brain tissues of the mice were harvested quickly under deep anesthesia. After washing with ice-cold normal saline, the brains were bisected in the midsagittal plane. One hemisphere was used for enzyme-linked immunosorbent assay (ELISA) kit analysis, while the opposite hemisphere was used for western blotting analysis. These samples were immediately stored at -80°C until used.

On the other hand, for immunofluorescence analysis, 4 mice in each group were deeply anesthetized and perfused intracardially with normal saline, followed by 4% paraformaldehyde (PFA) solution in 0.1 M phosphate buffer (PB, pH 7.4). The brain tissues were collected, postfixed in 4% PFA for 24 h, and then dehydrated in 30% sucrose at 4°C for 2-3 days. Transverse sections of the brain tissue (20 *μ*m) were obtained using a cryostat (Leica CM1850, Leica Microsystems GmbH, Wetzlar, Germany), then transferred to gelatin-coated slides at 20°C before further processing.

### 2.9. Measurement of the Levels of A*β*_40_ and A*β*_42_ in the Brains of TgCRND8 Mice

The levels of A*β*_40_ and A*β*_42_ in the brain tissues of TgCRND8 mice were measured using commercial mouse A*β*_40_ (Cat. KMB3481, Invitrogen, USA) and mouse A*β*_42_ (Cat. KMB3441, Invitrogen, USA) ELISA kits according to the manufacturer's protocols. Briefly, the brain hemisphere was homogenized in 8 × volume of homogenization buffer (5 M guanidine-HCl diluted in 50 mM Tris (pH 8.0)) with 1 × protease inhibitor cocktail containing AEBSF (Cat. P2714, Sigma, USA). The homogenate was mixed with an orbital shaker for 4 h at room temperature. After centrifugation at 16,000 × g at 4°C for 20 min, the supernatants were collected and diluted with standard diluent buffer to an appropriate concentration. The diluted supernatants were added into the wells that precoated with mAb to NH2 terminus of A*β* and then incubated for 2 h at room temperature to bind the antigen. The mouse A*β* detection antibody solution and anti-rabbit IgG HRP solution were sequentially added after washing three times with 1 × wash buffer. The reaction was terminated by adding the stop solution. The absorbance was determined at 450 nm within 10 min using a FLUOstar OPTIMA microplate reader (BMG Labtech, Offenburg, Germany). The levels of A*β*_40_ and A*β*_42_ in the brain tissues were calculated using the standard curves and expressed as pg/mg protein.

### 2.10. Determination of Cytokines in the Brain Tissues of TgCRND8 Mice

The brain tissues (100 mg) of mice were homogenized in lysis buffer (150 mM NaCl, 50 mM Tris-HCl (pH 7.4), 1 mM Na_3_VO_4_, 0.5% NP40, 1 mM NaF, and 1 mM DTT). After incubating for 15 min on ice, the homogenates were centrifuged at 10,000 × g at 4°C for 30 min. The protein levels of interleukin-6 (IL-6, Cat. No: ab100712), interleukin-1beta (IL-1*β*, Cat. No: ab100704), tumor necrosis factor alpha (TNF-*α*, Cat. No: LS-F5192), and interleukin-10 (IL-10, Cat. No: ab100697) in the supernatants were measured using commercially available sandwich ELISA kits (LifeSpan BioSciences, Seattle, USA, and Abcam, Cambridge, UK, respectively) per the manufacturer's protocols. The levels of IL-6, IL-1*β*, TNF-*α*, and IL-10 were expressed as pg/mg protein.

### 2.11. Immunofluorescence Assay

Brain sections containing the cortex and hippocampus were blocked with 5% bovine serum albumin (BSA) at room temperature for 1 h, then incubated with the following primary antibodies against mouse antiglial fibrillary acidic protein (GFAP) (1 : 1000, Cat. C106874, Sigma, USA) and rabbit anti-ionized calcium-binding adaptor molecule 1 (Iba-1) (1 : 1000, Cat. 019-19741, Wako, Japan), which were used to detect astrocyte and microglia, respectively. After washing for 5 min × 3 times with 1 × PBS (0.01 M, pH 7.4), the sections were then incubated with secondary antibodies against Alexa Fluor™ 647 streptavidin (Cat. S21374, Invitrogen, USA) or Alexa Fluor 488 Goat anti-Rabbit IgG (H+L) (Cat. R37116, Invitrogen, USA) for 2 h at room temperature in the dark. After washing with 1 × PBS for 3 times, sections were mounted on gelatin-coated glass slides and cover-slipped with antifade mounting medium (Dako, Glostrup, Denmark) for microscopic examination. BSA rather than the primary antibody was used as a negative control.

Fluorescent images were obtained using a Zeiss fluorescence microscope (Zeiss, Gottingen, Germany) equipped with an ORCA-Flash 4.0 v2 digital CMOS camera (Hamamatsu Photonics, Iwata City, Japan). Images were determined using an unbiased computer-assisted Image J software (NIH, Bethesda, MD, USA). The mean areas of the GFAP-positive astrocytes and Iba-1-positive microglia in each restricted area were quantified based on the method described in a previous study [28].

### 2.12. Western Blotting Analysis

Cytoplasmic and nuclear proteins were isolated from 100 mg of frozen brain tissues using the nuclear and cytosolic protein extraction kit (Chemicon, Temecula, CA, USA). Protein concentrations were determined using the BCA Protein Assay Kit. The protein samples were separated by sodium dodecyl sulfate-polyacrylamide (SDS-PAGE) at 80 V for 2 h. The separated proteins were transferred to polyvinylidene difluoride membrane (PVD) membranes using a transblotting apparatus (Bio-Rad Laboratories, USA) at 15 V for 30 min. The membranes were blocked with 5% (*w*/*v*) nonfat milk in TBS-T (Tris-buffer saline containing 0.1% Tween-20) at room temperature for 2 h. Subsequently, the membranes were incubated with an appropriate amount of primary antibodies against insulin-degrading enzyme (IDE) and presenilin-1 (PS-1) (Santa Cruz), *β*-site APP-cleaving enzyme-1 (BACE-1) and anterior pharynx-defective-1 (APH-1) (Sigma), neprilysin (NEP) (R&D Systems), p-APP (Thr688), postsynaptic density protein 93 (PSD93), postsynaptic density protein 95 (PSD95), synapsin 1 (SYN 1), synaptotagmin-1 (SYT 1), synaptophysin (SYN), nuclear factor kappa-B (NF-*κ*B) p65, p-NF-*κ*B p65, phosphor-glycogen synthase kinase-3*β* (p-GSK-3*β*), GSK-3*β*, phosphor-Akt (p-Akt), Akt (Cell Signaling Technology Inc., Beverly, MA, USA), and *β*-actin (Santa Cruz Biotechnology Inc., USA), respectively, at 4°C overnight. Next, the membranes were washed with TBS-T three times and probed with horseradish peroxidase-conjugated secondary antibodies for 1 h at room temperature. Finally, the membranes were washed with TBS-T three times before the protein bands were determined by the ECL western blotting detection reagents (Amersham Biosciences, Buckinghamshire, UK). The intensity of each band was quantified using Image J software.

### 2.13. Statistical Analysis

All data were presented as the mean ± SEM. Multiple group comparisons were analyzed to detect intergroup differences using one-way ANOVA followed by post hoc Bonferroni's test. GraphPad Prism software (Version 5, GraphPad Software, Inc., CA, USA) was used for the statistical analysis. A difference was considered statistically significant when *p* < 0.05.

## 3. Results

### 3.1. Effects of MN on the Locomotor Activity of TgCRND8 Mice

**Figure 3:**
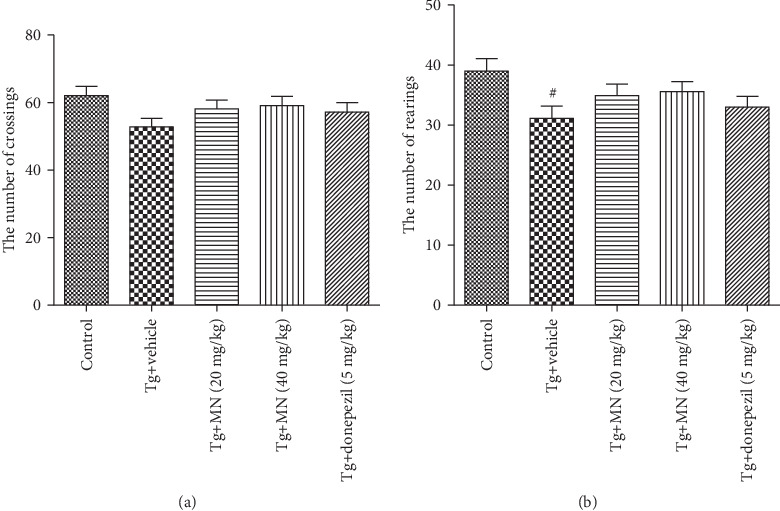
Effects of MN on the number of crossings (a) and rearings (b) in TgCRND8 mice as evaluated by the OPFT. Data were expressed as mean ± SEM (*n* = 10).^#^*p* < 0.05 when compared with the WT control.

Ten mice for each group were used to perform the open-field test. The number of rearings significantly decreased in TgCRND8 mice in the open-field test, while the number of crossings did not change markedly, as compared with the WT control group. Treatment with MN (20 and 40 mg/kg) or donepezil (5 mg/kg) did not significantly affect the number of rearings and crossings of TgCRND8 mice in the open-field test, as compared with the Tg vehicle control group (Figures 3(a) and 3(b)).

### 3.2. Effects of MN on the Recognition Impairment of TgCRND8 Mice

**Figure 4:**
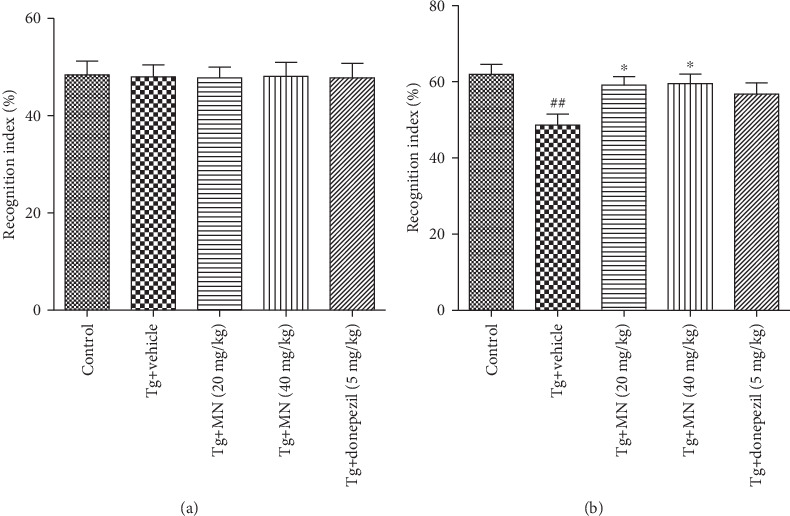
Effects of MN on the recognitive impairment in TgCRND8 mice as evaluated by the NORT. The recognition indices in the training session (a) and the recognition session (b) of the NORT. Data were expressed as mean ± SEM (*n* = 10). ^##^*p* < 0.01 when compared with the WT control; ^∗^*p* < 0.05 when compared with the Tg vehicle control.

The NORT is designed to measure the spontaneous preference of rodents to explore an unfamiliar object rather than a familiar object and is usually used to explore the recognition potential of the mice. Ten mice for each group were used to conduct the NORT. As shown in Figures 4(a) and 4(b), the recognition index was not significantly changed (*F*(4, 45) = 0.009, *p* > 0.05) in the training session of the NORT (Figure 4(a)). The recognition index was significantly different among various groups (*F*(4, 45) = 3.720, *p* < 0.05) in the recognition session of the NORT (Figure 4(b)). The recognition index in the TgCRND8 mice was significantly lower (*p* < 0.01) (Figure 4(b)) as compared with the WT control group. The mice treated with MN (20 and 40 mg/kg) exhibited a higher recognition index (*p* < 0.05 for both) in the recognition session of the NORT (Figure 4(b)) as compared with the Tg vehicle control group. These results suggest that MN could ameliorate the recognition impairments in TgCRND8 mice.

### 3.3. Effects of MN on Learning and Memory Impairments in TgCRND8 Mice

**Figure 5:**
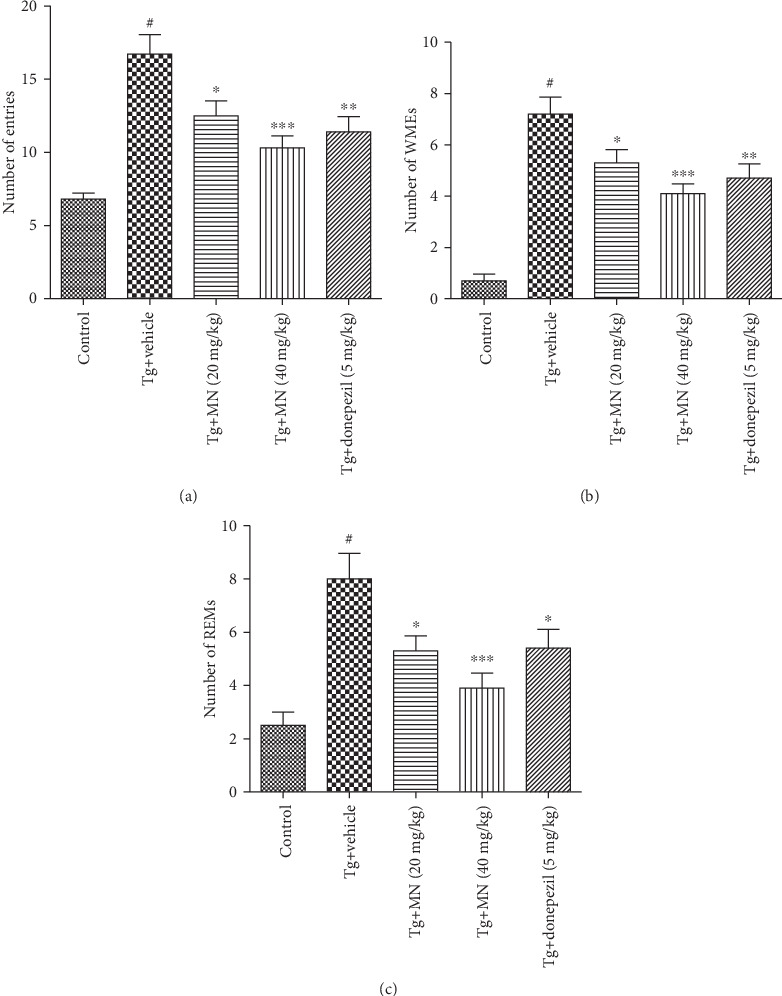
Effects of MN on the spatial learning and memory functions in TgCRND8 mice assessed by the RAMT. The total entries to complete the task test (a), the working memory errors (WMEs) (b), and the reference memory errors (RMEs) (c). Data were expressed as mean ± SEM (*n* = 10). ^#^*p* < 0.001 when compared with the WT control; ^∗^*p* < 0.05, ^∗∗^*p* < 0.01, and ^∗∗∗^*p* < 0.001 when compared with the Tg vehicle control.

The effects of MN on spatial learning and memory deficits were determined using the RAMT. Ten mice for each group were used to perform the RAMT. As shown in Figure 5(a), the number of total entries was significantly elevated (*F* (4, 45) = 13.410, *p* < 0.001) in the Tg vehicle group when compared to the WT group. The mice treated with MN (20 and 40 mg/kg) markedly decreased the number of total entries (*p* < 0.05 and *p* < 0.001, respectively) when compared to the Tg vehicle control group. Donepezil (5 mg/kg) treatment also markedly attenuated the number of total entries (*p* < 0.01) when compared to the Tg vehicle control.

The effects of MN on the numbers of WMEs and RMEs were shown in Figures 5(b) and 5(c), respectively. The results demonstrated that the numbers of WMEs (*F* (4, 45) = 22.870, *p* < 0.001) and RMEs (*F* (4, 45) = 9.041, *p* < 0.001) were effectively elevated in the Tg vehicle group when compared to the WT group. The mice treated with MN (20 and 40 mg/kg) significantly attenuated the numbers of WMEs (*p* < 0.05 and *p* < 0.001, respectively) and RMEs (*p* < 0.05 and *p* < 0.001, respectively) when compared to the Tg vehicle control group. Donepezil (5 mg/kg) treatment also obviously reduced the number of WMEs (*p* < 0.01) and RMEs (*p* < 0.05) when compared to the Tg vehicle control.

### 3.4. Effects of MN on the A*β* Deposition and APP Processing in the Brain Tissues of TgCRND8 Mice

**Figure 6:**
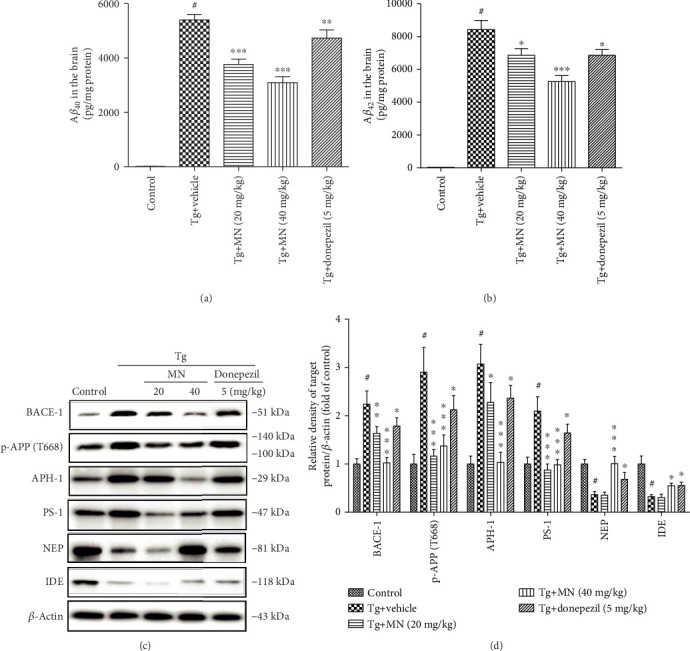
Effects of MN on the A*β* deposition and APP processing in the brain tissues of TgCRND8 mice. (a, b) The levels of A*β*_40_ and A*β*_42_ in the brain tissues of TgCRND8 mice were measured using ELISA kits. (c, d) The APP processing in the brain tissues of TgCRND8 mice were determined by western blot. Data were expressed as *mean* ± *SEM* (*n* = 3–6). ^#^*p* < 0.001 when compared with the WT control; ^∗^*p* < 0.05, ^∗∗^*p* < 0.05, and ^∗∗∗^*p* < 0.001 when compared with the Tg vehicle control.

The brain tissues from six mice for each group were used to determine the levels of A*β*_40_ and A*β*_42_. As shown in Figures 6(a) and 6(b), the protein levels of A*β*_40_ (*F* (4, 25) = 101.200, *p* < 0.001) and A*β*_42_ (*F* (4, 25) = 72.800, *p* < 0.001) were significantly higher in the brain of TgCRND8 mice when compared to the WT control. Treatment with MN (20 and 40 mg/kg) markedly reduced the protein levels of A*β*_40_ (*p* < 0.001 for both) and A*β*_42_ (*p* < 0.05 and *p* < 0.001, respectively) in the brain tissues of TgCRND8 mice, as compared to the WT control. Similarly, treatment with donepezil (5 mg/kg) also significantly decreased the levels of A*β*_42_ in the brain of TgCRND8 mice (*p* < 0.05).

The brain tissues from three mice for each group were used to measure the protein expressions of APP processing. As shown in Figures 6(c) and 6(d), the protein levels of BACE-1 (*F* (4, 10) = 28.180, *p* < 0.001), p-APP (T668) (*F* (4, 10) = 20.590, *p* < 0.001), APH-1 (*F* (4, 10) = 25.940, *p* < 0.001), and PS-1 (*F* (4, 10) = 24.920, *p* < 0.001) in the brain tissues of TgCRND8 mice were significantly increased, while the protein expressions of NEP (*F* (4, 10) = 25.630, *p* < 0.001) and IDE (*F* (4, 10) = 28.690, *p* < 0.001) were markedly decreased, when compared with the WT group. Treatment with MN (20 mg/kg) markedly suppressed the protein expressions of BACE-1 (*p* < 0.01), p-APP (*p* < 0.001), APH-1 (*p* < 0.05), and PS-1 (*p* < 0.001) but did not alter the protein expressions of NEP and IDE in the brain tissues of TgCRND8 mice when compared with the Tg vehicle group. On the other hand, treatment with MN (40 mg/kg) significantly inhibited the protein expressions of BACE-1 (*p* < 0.001), p-APP (*p* < 0.001), APH-1 (*p* < 0.001), and PS-1 (*p* < 0.001) but significantly enhanced the protein expressions of NEP (*p* < 0.001) and IDE (*p* < 0.001) in the brain tissues of TgCRND8 mice, as compared to the WT control group. Similarly, treatment with donepezil (5 mg/kg) significantly inhibited the protein expressions of BACE-1 (*p* < 0.05), p-APP (T668) (*p* < 0.05), and PS-1 (*p* < 0.001) but markedly elevated the protein expressions of NEP (*p* < 0.05) and IDE (*p* < 0.05) in the brain tissues of TgCRND8 mice as compared to the WT control group.

### 3.5. Effects of MN on the Astrocytes and Microglia in the Hippocampus and Cerebral Cortex of TgCRND8 Mice

**Figure 7:**
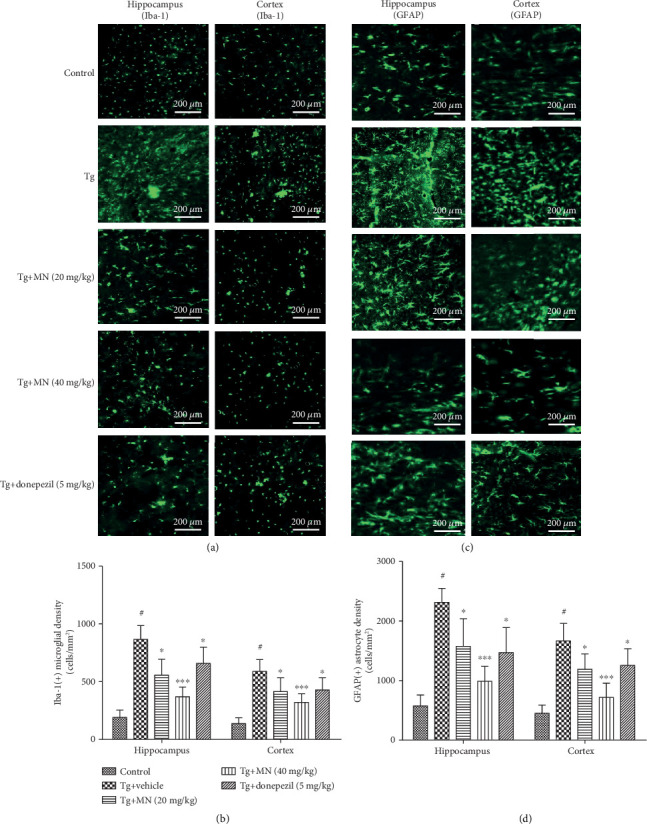
Effects of MN on the Iba1-positive microglia and GFAP-positive astrocytes in the hippocampus and cerebral cortex of TgCRND8 mice. (a, b) Microglial density was quantified by dividing the number of microglia by the area of the region of interest (cells/mm^2^), and the microglial density in the hippocampus and cerebral cortex was measured using the number of Iba-1-positive microglial cells. (c, d) Astrocyte density was quantified by dividing the number of astrocytes by the area of the region of interest (cells/mm^2^), and the astrocyte density in the hippocampus and cerebral cortex was determined by means of the number of GFAP-positive astrocyte cells. Data were expressed as mean ± SEM (*n* = 4). ^#^*p* < 0.001 when compared with the WT control; ^∗∗∗^*p* < 0.001 when compared with the Tg vehicle control.

The brain tissues from four mice for each group were used to evaluate the microglia and astrocytes in the hippocampus and cerebral cortex of the mice. As shown in Figures 7(a) and 7(b), a significant increase of microglial density was observed in the hippocampus (*F* (4, 15) = 26.580, *p* < 0.001) and cerebral cortex (*F* (4, 15) = 15.460, *p* < 0.001) of TgCRND8 mice when compared with the WT group. Treatment with MN (20 and 40 mg/kg) significantly decreased the microglial density in the hippocampus (*p* < 0.01 and *p* < 0.001, respectively) and cerebral cortex (*p* < 0.05 and *p* < 0.001, respectively) of TgCRND8 mice when compared with the Tg vehicle control. Similarly, treatment with donepezil (5 mg/kg) also effectively ameliorated the microglial density in the hippocampus (*p* < 0.05) and cerebral cortex (*p* < 0.05) of TgCRND8 mice.

Figures 7(c) and 7(d) revealed a marked increase of the astrocyte density in the hippocampus (*F* (4, 15) = 19.770, *p* < 0.001) and cerebral cortex (*F* (4, 15) = 19.240, *p* < 0.001) of TgCRND8 mice, when compared with the WT group. Treatment with MN (20 and 40 mg/kg) significantly attenuated the astrocyte density in the hippocampus (*p* < 0.01 and *p* < 0.001, respectively) and cerebral cortex (*p* < 0.05 and *p* < 0.001, respectively) of TgCRND8 mice when compared with the Tg vehicle control. Similarly, treatment with donepezil (5 mg/kg) also significantly reduced the astrocyte density in the hippocampus (*p* < 0.05) and cerebral cortex (*p* < 0.05) of TgCRND8 mice.

### 3.6. Effects of MN on the Levels of IL-6, IL-1*β*, TNF-*α*, and IL-10 in the Brains of TgCRND8 Mice

**Figure 8:**
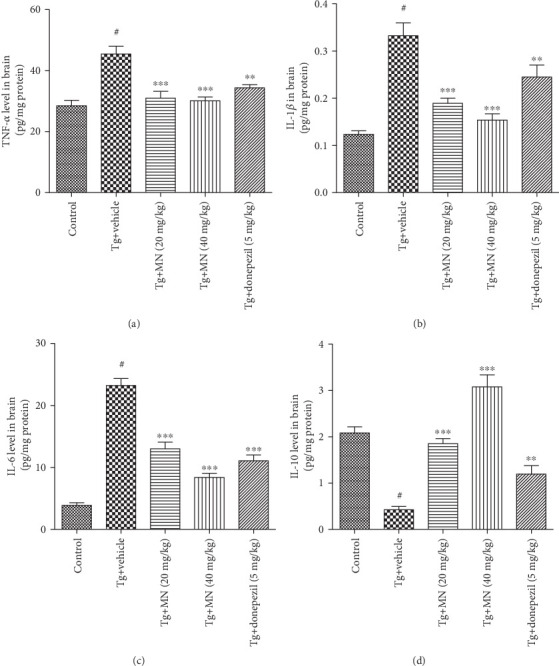
Effects of MN on the protein levels of TNF-*α* (a), IL-1s*β* (b), IL-6 (c), and IL-10 (d) in the brains of TgCRND8 mice. Data were expressed as mean ± SEM (*n* = 6). ^#^*p* < 0.001 when compared with the WT control; ^∗∗^*p* < 0.01 and ^∗∗∗^*p* < 0.001 when compared with the Tg vehicle control.

The brain tissues from six mice for each group were used to assay the protein levels of an inflammatory mediator. As shown in Figure 8, the protein levels of TNF-*α* (*F* (4, 25) = 12.950, *p* < 0.001) (a), IL-1*β* (*F* (4, 25) = 19.790, *p* < 0.001) (b), and IL-6 (*F* (4, 25) = 65.380, *p* < 0.001) (c) were significantly increased, while the release of IL-10 (*F* (4, 25) = 36.560, *p* < 0.001) (d) was markedly decreased in the brains of TgCRND8 mice, when compared to the WT control group. Treatment with MN (20 and 40 mg/kg) markedly decreased the protein productions of TNF-*α* (*p* < 0.001 for both), IL-1*β* (*p* < 0.001 for both), and IL-6 (*p* < 0.001 for both), while significantly elevated the release of IL-10 (*p* < 0.001 for both) in the brains of TgCRND8 mice, as compared to the Tg vehicle control group. Similarly, treatment with donepezil (5 mg/kg) also significantly reversed these changes of cytokines in TgCRND8 mice.

### 3.7. Effects of MN on the Synaptic Dysfunction in the Brains of TgCRND8 Mice

**Figure 9:**
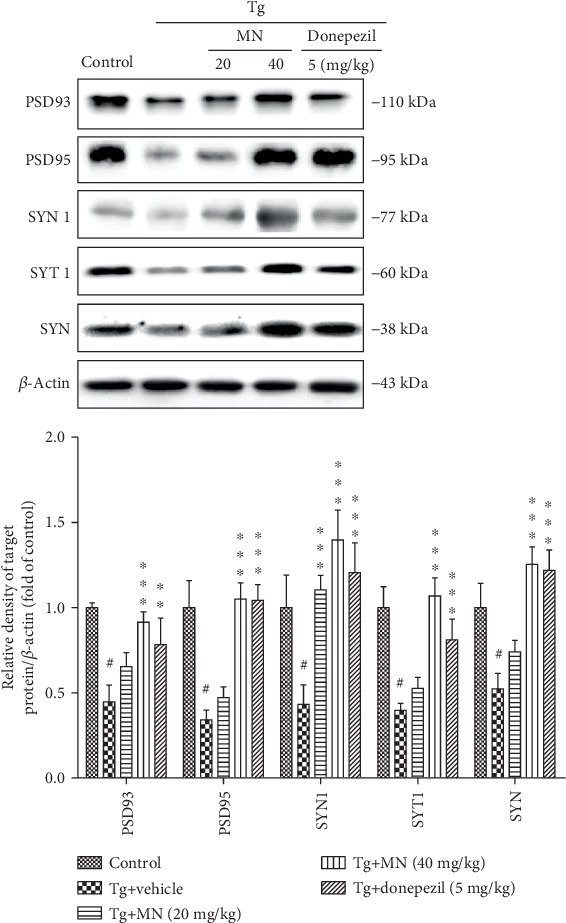
Effects of MN on the protein expressions of synapse proteins including PSD93, PSD95, SYN 1, SYT, and SYN in the brains of TgCRND8 mice. Data were expressed as mean ± SEM (*n* = 3). ^#^*p* < 0.001 when compared with the WT control; ^∗^*p* < 0.05, ^∗∗^*p* < 0.01, and ^∗∗∗^*p* < 0.001 when compared with the Tg vehicle control.

Synaptic dysfunction is an early event in AD patients and correlates well with cognitive impairment during the course of the disease [29]. To evaluate the effects of MN (20 and 40 mg/kg) on synaptic pathology, we examined the levels of PSD93, PSD95, SYN 1, SYT, and SYN in the brains of TgCRND8 mice (*n* = 3) (Figure 9). The results demonstrated that the protein levels of PSD93 (*F* (4, 10) = 15.890, *p* < 0.001), PSD95 (*F* (4, 10) = 35.750, *p* < 0.001), SYN 1 (*F* (4, 10) = 16.940, *p* < 0.001), SYT 1 (*F* (4, 10) = 27.240, *p* < 0.001), and SYN (*F* (4, 10) = 25.300, *p* < 0.001) were significantly decreased in the brains of TgCRND8 mice as compared with the WT control group. Treatment with MN (20 mg/kg) markedly elevated the protein expressions of SYN 1 (*p* < 0.01) in the brains of TgCRND8 mice. Interestingly, MN (40 mg/kg) significantly enhanced the protein expressions of PSD93 (*p* < 0.001), PSD95 (*p* < 0.001), SYN 1 (*p* < 0.001), SYT 1 (*p* < 0.001), and SYN (*p* < 0.001) in the brains of TgCRND8 mice as compared with the Tg vehicle control. Similarly, donepezil (5 mg/kg) obviously increased the protein expressions of PSD93 (*p* < 0.01), PSD95 (*p* < 0.001), SYN 1 (*p* < 0.001), SYT 1 (*p* < 0.001), and SYN (*p* < 0.001) in the brains of TgCRND8 mice, as compared with the Tg vehicle control.

### 3.8. Effects of MN on the NF-*κ*B and PI3K/Akt/GSK-3*β* Pathways in the Brains of TgCRND8 Mice

**Figure 10:**
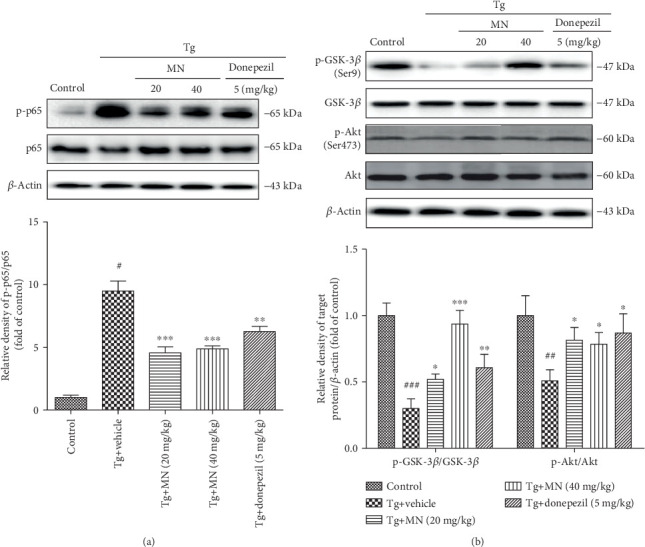
Effects of MN on the NF-*κ*B (a) and PI3K/Akt/GSK-3*β* (b) signaling pathways in the brain tissues of TgCRND8 mice. Data were expressed as mean ± SEM (*n* = 3). ^#^*p* < 0.001 when compared with the WT control; ^∗^*p* < 0.05, ^∗∗^*p* < 0.01, and ^∗∗∗^*p* < 0.001 when compared with the Tg vehicle control.

The NF-*κ*B pathway plays a central role in regulating inflammatory responses. The brain tissues of three mice for each group were used to determine the protein expressions of the NF-*κ*B and PI3K/Akt/GSK-3*β* pathways. As shown in Figure 10(a), the ratio of p-NF-*κ*B p65/NF-*κ*B p65 (*F* (4, 10) = 41.350, *p* < 0.001) was significantly accentuated in the brains of TgCRND8 mice when compared with the WT control. However, treatment with MN (20 and 40 mg/kg) markedly reduced the ratio of the protein expression of p-NF-*κ*B p65/NF-*κ*B p65 (*p* < 0.001 for both) in the brains of TgCRND8 mice, when compared with the Tg vehicle control. Treatment with donepezil (5 mg/kg) also conspicuously suppressed the ratio of the protein expression of p-NF-*κ*B p65/NF-*κ*B p65 (*p* < 0.001) in the brains of TgCRND8 mice when compared with the Tg vehicle control.

The results shown in Figure 10(b) revealed that there was a significant decrease in the protein expressions of p-GSK-3*β* (Ser9) (*F* (4, 10) = 35.090, *p* < 0.001) and p-Akt (Ser473) (*F* (4, 10) = 7.257, *p* < 0.01) in the brain tissues of TgCRND8 mice, when compared with the WT group. Treatment with MN (20 and 40 mg/kg) markedly enhanced the ratios of the protein expressions of p-GSK-3*β* (Ser9)/GSK-3*β* (*p* < 0.05 and *p* < 0.001, respectively) and p-Akt (Ser473)/Akt (*p* < 0.05 for both) in the brain tissues of TgCRND8 mice when compared with the Tg vehicle group. Similarly, treatment with donepezil (5 mg/kg) also obviously increased the ratios of the protein expressions of p-GSK-3*β* (Ser9)/GSK-3*β* (*p* < 0.01) and p-Akt (Ser473)/Akt (*p* < 0.05) in the brain tissues of TgCRND8 mice when compared with the Tg vehicle group.

## 4. Discussion

Previous studies have demonstrated that MN administration exerts significant therapeutic action on AD [17, 19]. In the present study, our data for the first time revealed that MN could ameliorate the cognitive deficits in TgCRND8 transgenic mice via inhibition of neuroinflammation and synaptic dysfunction through modulating the PI3K/Akt/GSK-3*β* and NF-*κ*B pathways. Donepezil, an acetylcholinesterase inhibitor approved by the United States Food and Drug Administration for the treatment of mild to moderate AD, was used as a positive control in this study. However, it has undesirable side effects in AD patients such as nausea, vomiting, diarrhea, dizziness, drowsiness, and trouble sleeping [30]. In this study, our results indicated that donepezil could suppress the neuroinflammation and synaptic dysfunction to improve cognitive deficits in TgCRND8 transgenic mice via regulating the PI3K/Akt/GSK-3*β* and NF-*κ*B pathways. Interestingly, MN at a dose of 40 mg/kg exerted more potent effects than donepezil in the improvement of cognitive deficits, inhibition of neuroinflammation, A*β* deposition, and synaptic dysfunction in TgCRND8 mice. Moreover, it has been shown that the 50% lethal dose (LD_50_) values of MN and donepezil by oral administration in mice are about 2200 mg/kg and 45.2 mg/kg, respectively, [31, 32], suggesting that the toxicity of MN is about 50 times less than donepezil, thus is a safer plant-derived compound for AD treatment.

A*β* deposition in the brain is one of the major hallmarks of AD pathogenesis. In the amyloidogenic pathway, APP is primarily processed by *β*- and *γ*-secretases to produce A*β*. BACE-1, a key *β*-secretase, is essential for initiating A*β* production [33]. *γ*-Secretase involves a large proteinase complex comprising at least four major protein ingredients such as APH-1 and PS-1 [34]. Both IDE and NEP are the two major A*β*-degrading enzymes in the APP processing [35]. Thus, inhibition of the activities of *β*-secretase or *γ*-secretase or enhancement of the A*β*-degrading enzymatic activities may help to reduce the A*β* deposition. The results of our present study revealed that MN treatment significantly inhibited the protein expressions of BACE-1, p-APP (T668), APH-1, and PS-1, as well as the protein levels of A*β*_40_ and A*β*_42_, while markedly enhanced the protein expression of NEP and IDE in the brains of TgCRND8 mice. These results strongly suggested that MN mitigated the A*β* deposition via inhibiting the activities of *β*- and *γ*-secretases and enhancing the activities of A*β*-degrading enzymes in the brains of TgCRND8 mice.

A growing body of evidence has revealed that neuroinflammation plays an important role in AD pathology [36, 37]. Neuroinflammation in AD is considered to be primarily driven by microglial cells [38]. Importantly, the oligomers and fibrils of A*β* are capable of priming microglial cells through elevating the production of inflammatory cytokines (TNF-*α*, IL-6, and IL-1*β*) and suppressing anti-inflammatory mediators (IL-4 and IL-10), thereby promoting the activation of microglia [39]. Overactivation and gliosis of microglia have been found to play critical roles in AD pathology [40]. The activated microglia can be polarized into divergent M2 or M1 phenotypes and secrete corresponding anti-inflammatory or proinflammatory mediators. Overactivation of the M1 microglia phenotype leads to the secretion of proinflammatory cytokines, subsequently inducing neurotoxicity and neurodegeneration [41–43]. In contrast, the M2 phenotype microglia enhance the release of anti-inflammatory mediators to promote tissue repair and extracellular matrix reconstruction [43]. An abnormal elevation of proinflammatory cytokines has been reported to be closely associated with memory impairment and affective disorders such as depression and anxiety [44]. Consequently, inhibition of neuroinflammation is crucial for the treatment of AD. MN has been reported to inhibit neuroinflammation in mice exhibiting depressive-like behaviors [45]. To confirm the impacts of MN on M1/M2 microglia polarization, we measured the levels of specific proinflammatory cytokines and anti-inflammatory mediators in the brains of TgCRND8 mice. Our results consistently demonstrated that MN treatment attenuated the release of proinflammatory cytokines such as IL-1*β*, IL-6, and TNF-*α*, while augmented the production of an anti-inflammatory mediator such as IL-10, indicating its anti-inflammatory potential. Interestingly, our present experimental results revealed that MN could attenuate the release of proinflammatory cytokines while accentuating the production of anti-inflammatory mediators, indicating that the anti-inflammatory activities of MN also contribute to its cognition-enhancing functions in TgCRND8 mice.

AD patients have a series of pathological characteristics, such as neurofibrillary tangles, senile plaques, and a massive loss of brain weight and volume, especially in the hippocampus, a region most closely associated with memory functions [46]. A synapse is a basic unit that transmits information among neurons and is under tight spatiotemporal regulation, and the aberrant function of synapses is strongly implicated in various neurological disorders such as AD [47]. Loss of functional synapses accompanied by learning and memory impairments is also apparent in various AD transgenic animal models [48–50]. Synaptic plasticity forms the molecular foundation of learning and memory in the central nervous system. As the structural function of cognition, synapse proteins such as PSD93, PSD95, SYN 1, SYT 1, and SYN play crucial roles in signaling conduction and learning and memory function. In the present study, we found that MN administration significantly increased the levels of some synaptic proteins including PSD93, PSD95, SYN 1, SYT 1, and SYN in the brains of TgCRND8 mice. Taken together, these results suggest that the preventive effects of MN treatment on the learning and memory impairments of TgCRND8 mice are at least partially associated with the improvement of the synaptic dysfunction.

The phosphoinositide 3-kinase/protein kinase B (PI3K/Akt) signaling pathway is important for sustaining the function of neurons. Increasing evidence reveals that the PI3K/Akt/GSK-3*β* pathway can be altered by A*β* deposit in the brains of AD patients. Dysfunction of the PI3K/Akt signaling pathway can increase the activity of GSK-3*β* and regulate the metabolism of A*β*, leading to the hyperphosphorylation and deposition of tau protein, thus contributing to the formation of neurofibrillary tangles in the AD brains [51]. The “GSK3 hypothesis in AD” suggests that the overactivation of GSK-3*β* is closely related to several features of the pathology of AD, including microglia-mediated inflammation, A*β* production, APP processing, tau phosphorylation, neuronal death, and cognitive deficits [52]. A recent study revealed that GSK-3*β* deletion in the dentate gyrus of mice suppressed hippocampal synaptic transmission and decreased the levels of synapse proteins such as PSD93 and SYN [53]. Thus, inhibition of GSK-3*β* has been found to be beneficial and provide a potential therapeutic target for neurodegenerative diseases [54]. A previous study demonstrated that MN could modulate the activation of Akt in SAMP8 mice [17]. Our present results indicated that MN markedly activated the Akt activity and inhibited the GSK-3*β* activation to maintain the synaptic plasticity and memory function of TgCRND8 mice.

**Figure 11:**
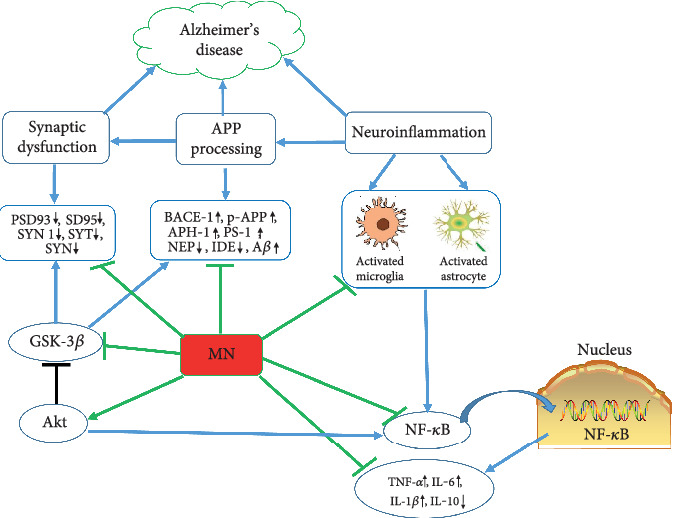
Schematic drawing depicting the molecular mechanisms associated with the cognitive deficits improving effects of MN on TgCRND8 mice. The results of this present study demonstrated that MN could inhibit the activation of microglia and astrocytes to reduce the A*β* deposit, as well as suppress the release of proinflammatory cytokines such as IL-1*β*, IL-6, and TNF-*α*, while promoting the production of the anti-inflammatory mediators such as IL-10. Moreover, MN increased the protein expressions of the functional synaptic proteins such as PSD93, PSD95, SYN 1, SYT 1, and SYN in the brains of TgCRND8 mice. Changes of the PI3K/Akt/GSK-3*β* and NF-*κ*B pathways also occurred in the brains of TgCRND8 mice. MN treatment reduced A*β* deposit, inhibited neuroinflammation, and reversed the synaptic deficits via regulating the PI3K/Akt/GSK-3*β* and NF-*κ*B pathways. These cellular actions of MN collectively contribute to the therapeutic effects on AD in TgCRND8 mice as evidenced by the improvement in the spatial learning and memory functions.

NF-*κ*B is a key nuclear transcription factor playing a cardinal role in the inflammatory response [55]. Under normal physiological conditions, the inactive NF-*κ*B bound to the inhibitory protein I*κ*B kinase and is located in the cytosol [56]. After stimulated by cellular stress, the NF-*κ*B complex is activated by dissociation from I*κ*B [57]. Activated NF-*κ*B is then translocated from the cytosol to the nucleus to promote the expression of downstream proinflammatory target genes [58]. More interestingly, the activation of inflammasome plays a critical role in the pathogenesis of AD, such as A*β* production and cognitive impairments, via modulating the chronic inflammatory response [59]. Therefore, inhibition of the activation of the NF-*κ*B pathway would be beneficial for the suppression of inflammatory processes. MN is widely used as an effective anti-inflammatory agent. A recent study showed that MN markedly inhibited the inflammatory responses stimulated by the fimbriae of Porphyromonas gingivalis via inhibition of the NF-*κ*B pathway in RAW264.7 macrophages [60]. MN also attenuated the NLRP3 inflammasome via inhibition of the NF-*κ*B signaling pathway on lupus nephritis in MRL/lpr mice [61]. Consistent with these previous studies, we also found MN treatment conspicuously alleviated neuroinflammation partially by inhibiting the NF-*κ*B pathway activation in the brains of TgCRND8 mice.  Figure 11 is a schematic drawing depicting the molecular mechanisms associated with the cognitive deficits improving the effects of MN on TgCRND8 mice.

## 5. Conclusions

In sum, our project has revealed for the first time that MN ameliorates the learning and memory impairments in TgCRND8 mice, and the neuroprotective effects of MN are attributed to (1) the suppression of A*β* deposition via inhibiting the activities of *β*- and *γ*-secretases, and the enhancement of the activities of A*β*-degrading enzymes, and (2) the inhibition of neuroinflammatory and synaptic dysfunction, partially via regulation of PI3K/Akt/GSK-3*β* and NF-*κ*B signaling pathways. Among the underlying mechanisms, the synaptic dysfunction inhibitory effect may be dominant in the anti-AD effects of MN. Therefore, more investigations are warranted to explore the anti-AD effects of MN targeting synaptic dysfunction modulation. We believe that MN is a promising naturally occurring constituent worthy of further development into anti-AD therapeutics.

## Data Availability

All data supporting the conclusions of this article are included in this article.

## Abbreviations

A*β*: Beta-amyloid
AD: Alzheimer's disease
APH-1: Anterior pharynx-defective-1
APP: Amyloid precursor protein
BACE-1: *β*-Site APP-cleaving enzyme-1
BBB: Blood-brain barrier
BSA: Bovine serum albumin
CMC-Na: Sodium carboxymethyl cellulose
ELISA: Enzyme-linked immunosorbent assay
IDE: Insulin-degrading enzyme
IL-6: Interleukin-6
IL-10: Interleukin-10
IL-1*β*: Interleukin-1beta
MN: Magnolol
NEP: Neprilysin
NF-*κ*B: Nuclear factor kappa-B
NORT: Novel objective recognition test
OFT: Open-field test
PCR: Polymerase chain reaction
PFA: Paraformaldehyde
PS-1: Presenilin-1
PSD93: Postsynaptic density protein 93
PSD95: Postsynaptic density protein 95
RAMT: Radial arm maze test
RMEs: Reference memory errors
SYN 1: Synapsin 1
SYN: Synaptophysin
SYT 1: Synaptotagmin-1
Tg: TgCRND8
TNF-*α*: Tumor necrosis factor alpha
WMEs: Working memory errors
WT: Wild type.
